# Recent Advances in Mathematical Methods for the Analysis of Biomedical Image

**DOI:** 10.1155/IJBI/2006/70578

**Published:** 2006-12-06

**Authors:** Guowei Wei

**Affiliations:** Department of Mathematics and Department of Electrical and Computer Engineering, Michigan State University, East Lansing, MI 48824, USA


Hindawi Publishing Corporation
International Journal of Biomedical Imaging
Volume 2006, Article ID 70578, Pages 1–1
DOI 10.1155/IJBI/2006/70578

*Editorial*

### Recent Advances in Mathematical Methods for the Analysis of Biomedical Image

**Guowei Wei**

*Department of Mathematics and Department of Electrical and Computer Engineering, Michigan State University, East Lansing, MI 48824, USA*

Received 10 September 2006; Accepted 10 September 2006

Copyright © 2006 Guowei Wei. This is an open access article distributed under the Creative Commons Attribution License, which permits unrestricted use, distribution, and reproduction in any medium, provided the original work is properly cited.

Image analysis, an interdisciplinary field where one witnesses the crossing contribution from engineers, scientists and mathematicians, is of paramount importance for a wide range of technologies, such as computer vision, pattern recognition, artificial intelligence, and biomedical imaging. During the last few years, there have been important advances in the development of the next generation of mathematical methods for image analysis. While progress in the fundamentals and applications has been rapid, many challenges remain. This special issue is a collection of high quality, peer-reviewed, original research papers illustrating recent progress and future directions in the area of mathematical analysis of biomedical images.

The editor of this special issue expresses his sincere thanks to the contributing authors and reviewers for making this publication possible and successful.

*Guowei Wei*

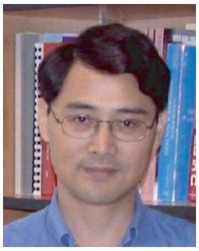

**Guowei Wei** received his Ph.D. degree from the University of British Columbia in 1996. With a postdoctoral fellowship Award from the NSERC of Canada, he did research work at the University of Houston, where he was appointed as a Research Assistant Professor in 1997. In 1998, he joined the Faculty of the National University of Singapore and was promoted to Associate Professor in 2001. In 2002, he relocated to Michigan State University, where he is currently an Associate Professor of Mathematics and Electrical and Computer Engineering. He has advised 10 doctoral students, 7 master students, and 8 postdoctoral fellows. He has authored or coauthored over 130 refereed journal papers. His current research interests include scientific and engineering computations, biomedical image analysis, controlling chaos and turbulence, mathematical modeling, and computation of protein structures and interactions.

